# Melatonin Protects Tobacco Suspension Cells against Pb-Induced Mitochondrial Dysfunction

**DOI:** 10.3390/ijms222413368

**Published:** 2021-12-13

**Authors:** Agnieszka Kobylińska, Małgorzata Maria Posmyk

**Affiliations:** Department of Plant Ecophysiology, Faculty of Biology and Environmental Protection, University of Lodz, 90-237 Łódź, Poland; agnieszka.kobylinska@biol.uni.lodz.pl

**Keywords:** BY-2 tobacco cells, caspase-like protease, cytochrome c, melatonin, mitochondria, programmed cell death

## Abstract

Recent studies have shown that melatonin is an important molecule in plant physiology. It seems that the most important is that melatonin effectively eliminates oxidative stress (direct and indirect antioxidant) and switches on different defence strategies (preventive and interventive actions) during environmental stresses. In the presented report, exogenous melatonin potential to protect *Nicotiana tabacum* L. line Bright Yellow 2 (BY-2) exposed to lead against death was examined. Analyses of cell proliferation and viability, the level of intracellular calcium, changes in mitochondrial membrane potential (*ΔΨm*) as well as possible translocation of cytochrome c from mitochondria to cytosol and subsequent caspase-like proteolytic activity were conducted. Our results indicate that pretreatment BY-2 with melatonin protected tobacco cells against mitochondrial dysfunction and caspase-like activation caused by lead. The findings suggest the possible role of this indoleamine in the molecular mechanism of mitochondria, safeguarding against potential collapse and cytochrome c release. Thus, it seems that applied melatonin acted as an effective factor, promoting survival and increasing plant tolerance to lead.

## 1. Introduction

Among the most significant soil contaminants resulting from both natural and manmade sources, heavy metals, and especially lead, are of prime importance due to their long-term toxicity effects [1]). Lead (Pb) can be found in dust, fumes, mists, vapours and in soil as minerals (PbCO_3_—cerussite, PbS—galena, PbSO_4_—anglesite) [1]. Lead is taken up via roots, along with water, or it can be absorbed from the air via shoots and foliage [2]. Unfortunately, plant roots are not highly selective and absorb excessively accumulated lead. It affects mineral uptake, inducing imbalances in essential and trace elements resulted from toxic ion competitions with macro- and microelements and their replacement in various biologically active substances [3,4]. Thus, the decrease of P, K, Ca, Cu, Fe, Mn and Zn content in plant tissue, as a result of a possible blockage of the transporter proteins by lead, is observed [2,3,5].

Moreover, a higher level of lead in plant tissues leads to alterations in photosynthesis, revealed as a limitation of net CO_2_ assimilation rate, decline of chlorophylls concentration due to the reduction of chlorophyll synthesis, and their degradation as well as destabilization of chloroplast structure [5,6].

The latest literature data indicated that water-extractable lead (soils leachates), similarly to other heavy metals, are most likely involved in human DNA damage [7]—it is potentially mutagenic, capable of inhibiting DNA synthesis or interfering with DNA repair. The cytotoxic mechanisms of lead in plants are not entirely understood. It has been reported that lead as a non-redox heavy metal can induce enhanced accumulation of reactive oxygen species (ROS), contributing to the strong oxidative stress generation and causing lipid peroxidation, membrane dysfunction, protein damage, DNA injuries and cell death [8,9].

Its toxic concentration inhibits the activity of key enzymes, e.g., acid phosphatase, esterases, peroxidases, and malic dehydrogenase, by reacting with their sulfhydryl groups. Moreover, in BY-2 tobacco suspension cells, lead was the cause of the so-called DNA laddering, which is one of the signs of programmed cell death (PCD) [10].

PCD plays a key role in plant development and responses to environmental stresses. In multicellular eukaryotes, it occurs as part of normal development or maintenance of tissue homeostasis and is therefore one of the critical mechanisms for survival [11]. Plant cells, similar to cells from other kingdoms, have the ability to self-destruct in a genetically controlled way, and the identification of key regulators of plant PCD has been the focus of many studies [12,13]. The signalling cascades of animal PCD are well examined in comparison to plants and have clearly defined molecular subroutines, as was described by the nomenclature committee on cell death [14]. However, despite the vast evolutionary distance between mammals and plants, there are some common features of their PCD, including: increased formation of vesicles, cytoplasmic condensation, nuclear condensation, DNA degradation into mono- or oligonucleosomal 140–180 [15] or into 50 kbp fragments [16] fragments as well as translocation of cytochrome c (Cyt c) from mitochondria to the cytosol [17,18]. During PCD, plant release of Cyt c can occur as a result of many stimuli such as D-mannose, menadione, heat, sulphated lentinan and ROS [19,20,21,22,23], whereas the production of endogenous ROS (low level as signalling molecules) is often affected by fluctuations in intracellular calcium ion (Ca^2+^) concentration. It is known that Ca^2+^ and calmodulin are the ubiquitous intracellular second messengers regulating a large array of biological processes in plants. Specificity of calcium-based signalling processes result from spatio-temporal dynamics and the origin of calcium signals [24].

Thus, variation of intracellular Ca^2+^/calmodulin concentration could trigger the PCD process in plants [25,26]. It was shown that in the interaction between pathogen and plant, an increase of Ca^2+^-activated ROS generation (high level as anti-pathogen strategy) and induced hypersensitive response (HR)-like cell death [26]. In this case, an increase of cytoplasmic Ca^2+^ concentration in plant cells seems to be an essential prerequisite for stimulating plant emergency response and inducing the production of resistant compounds such as ROS [26,27]. According to Petrosillo et al. [28,29] mitochondrial-induced ROS production promotes translocation of Cyt c from mitochondria to cytosol and may occur by a two-step process wherein Cyt c is first dissociated from cardiolipin via cardiolipin peroxidation, which is then released into the extramitochondrial environment followed by permeabilization of the outer membrane, probably by interaction with voltage dependent anion channels [28,29]. However, the function of cytoplasmic Cyt c is still discussed, since Vacca et al. [30] found that Cyt c release depended on ROS production and takes part in activation of the caspase-like cascade, but it is does not always lead to PCD. Vacca revealed that in early steps after PCD induction, an immediate production of ROS occurs that promotes Cyt c release from intact mitochondria. The caspase-like proteases play no role in this phase, essentially being inactive. In the later phase after PCD induction, the released Cyt c contributes to activation in caspase-like proteases, resulting in degradation of Cyt c and cell death [30]. However, Martínez-Fábregas et al. [18] indicated that extra-mitochondrial Cyt c had a double role in causing living cells to die by activation of the proapoptotic pathway as well as by inhibiting pro-survival factors, including SET protein (which acts as an inhibitor of p53 acetylation and blocks both p53-mediated cell cycle arrest and apoptosis after stress) or luminal binding protein 1 and 2 (BiP1 and BiP2), whose overexpression increased cell tolerance to endoplasmic reticulum stress [18,31]. 

To reduce the negative impact of environmental stresses, including lead pollution and defending cells against damage, the best solution may be biostimulators, non-toxic substances of natural origin that improve and stimulate plant life processes (phytostimulators) differently than fertilizers or phytohormones, e.g., improving plant tolerance and protecting them against harmful factors [32]. It is known that melatonin (N-acetyl-5-methoxytryptamine) seems to have great biostimulatory potential. This indolamine may act as a factor, fortifying cells against multiple stresses such as cold [33], copper [34], cadmium [35], lead [9,10], high temperature [36], salt [37], pressure osmotic [38], drought stresses [39] and pathogen infection [40]. Melatonin also redirects carbohydrate metabolism during sugar starvation and induces gluconeogenesis to obtain basic energy substrates, which is another defence strategy to overcome adverse environmental conditions [41]. Analysis of proteome in corn embryo after hydropriming with melatonin led to identification of more than twenty additional proteins, among which are identified anti-stress proteins as well as ones responsible for defence and detoxification [42]. Melatonin is the only antioxidant that can induce the cascade of reaction, in which both melatonin and its metabolites, e.g., cyclic-3-hydroxymelatonin, 2-hydroxylmelatonin and especially N1-acetyl-N2-formyl-5-methoxykynuramine, function as ROS scavengers.

Since melatonin has amphiphilic characteristics, it may act as a hydrophilic and hydrophobic antioxidant. This fact, together with small-sized melatonin, makes it particularly able to migrate easily between cell compartments in order to protect them against excessive ROS. Maintenance the ROS homeostasis in plants is crucial because, as reported by Bolduc and Brisson as well as Kawai-Yamada et al. [43,44], bursts of oxidative metabolism leading to generation of ROS are one of the earliest events in PCD, induced by harmful environmental conditions in plant cells. This suggests that high levels of ROS mediate the signal network for defence gene induction, e.g., *hsp*, *lea*, *cor*, *ecs* [45], or for PCD of selected cells.

One of the primary sites of ROS formation and the first target of oxidative stress are mitochondria. This is why mitochondria require some mechanisms that reduce oxidative stress levels in order to protect against abiotic or biotic stresses. Among many specific ROS-scavenging tools, melatonin seems to be a mitochondrial-targeted antioxidant. The current data suggest that melatonin is not only taken up by mitochondria, but these organelles, which is highly probable, also produce melatonin [46,47,48]. Our previous studies revealed that melatonin regulates ROS generation by modulation expression of Bax inhibitor-1, a protein that functions as a Ca^2+^/H^+^ antiporter.

We demonstrated that significant increases in cells viability and beneficial effects of exogenous melatonin on Pb-exposed BY-2 cells are correlated with drastically decreasing in O_2_ ^•-^ and H_2_O_2_ contents and with changes in the expression of BI-1 protein—an ancient regulator of plant cell death. However, there is still relatively little knowledge about the mechanisms of action of melatonin on cytological level in plants during preservation against death.

The aim of this study was to further elucidate the mechanisms underlying *Nicotiana tabacum* L. (cv. Bright Yellow 2; BY-2) cells’ resistance to exposure to lead by pre-treatment with melatonin, with particular emphasis on several signalling events in mitochondria. The selection of the in vitro plant model to be tested was dictated by the homogeneity of the material (suspension of undifferentiated cells) and the possibility of multiple repetitions of the experiments under the same strictly controlled conditions.

Obtained results provide strong evidence that lead induces cell death through dysfunction of mitochondria whilst melatonin induces biological responses to fortifying cells against environmental stress. The findings show that melatonin significantly limited the negative effects of this heavy metal and acted as a biostimulating, pro-survival factor.

## 2. Results

### 2.1. Cell Growth and Viability after Lead Treatment

**Figure 1:**
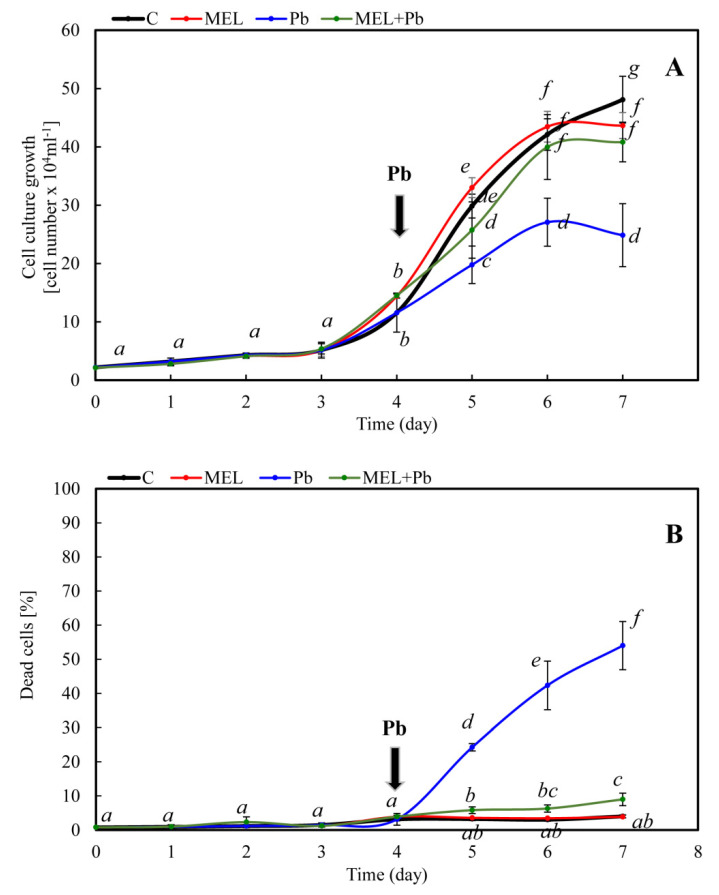
Kinetics of cell proliferation (**A**) and mortality (**B**) of BY-2 tobacco cells during conducted culture (0–7 days). Cell variants: **C**—BY-2 cells cultured on LS medium (the control variant); MEL—BY-2 cells cultured on LS medium with 200 nM of melatonin added from the beginning of the culture; **Pb**—BY-2 cells cultured on LS medium with 15 µM Pb^2+^ added on the fourth day of the culture and MEL+Pb—BY-2 cells cultured on LS medium with melatonin added from the start of the culture and with Pb^2+^ added on the fourth day of culture. (**A**) Proliferation of ANOVA results: Variant (C, MEL, Pb, MEL + Pb) F_(3; 84)_ = 45.2 *p* < 0.00001; Day of culture (0, 1, 2, 3, 4, 5, 6, 7) F_(7; 84)_ = 722.7 *p* < 0.00001; and interaction Variant x Day of culture F_(21; 84)_ = 12.6 *p* < 0.00001. (**B**) Mortality ANOVA results: Variant (C, MEL, Pb, MEL + Pb) F_(3; 71)_ = 334.7 *p* < 0.00001; Day of culture (0, 1, 2, 3, 4, 5, 6, 7) F_(7; 71)_ = 165.6 *p* < 0.00001; and interaction Variant x Day of culture F_(21; 71)_ = 98.4 *p* < 0.00001.

Incubation of BY-2 cells with melatonin prior to lead exposure protected cells from death and slightly improved cell proliferation, especially in the *log* phase of growth. Growth rates of the C and MEL cells were similar during all culture times. Lead stress was triggered by the addition of lead on the fourth day and immediately resulted in significant inhibition of tobacco cell proliferation; see variant Pb (Figure 1A). However, proliferation of the MEL + Pb cells was 30–40% higher in comparison to those that were lead-treated and not primed with melatonin—Pb (Figure 1A). This effect remained until the end of the BY-2 cell culture.

Conversely, the protective role of melatonin was evaluated by cell mortality assessment in all experimental samples. Methylene blue staining evidenced that culture medium supplementation with melatonin did not result in cell death acceleration. Surprisingly, obtained results revealed a comparable level of cell viability between lead-untreated cells (C and MEL) and lead exposed but primed with melatonin ones (MEL + Pb) (Figure 1B).

Moreover, received data showed inhibitory effect of melatonin on cell death induced by lead. At the end of the *log* phase (the sixth day of culture; the second day after lead administration), mortality of BY-2 cells in Pb variants was 85% higher than in the MEL + Pb variant (Figure 1B).

### 2.2. Measurements of Intracellular Calcium Level

**Figure 2:**
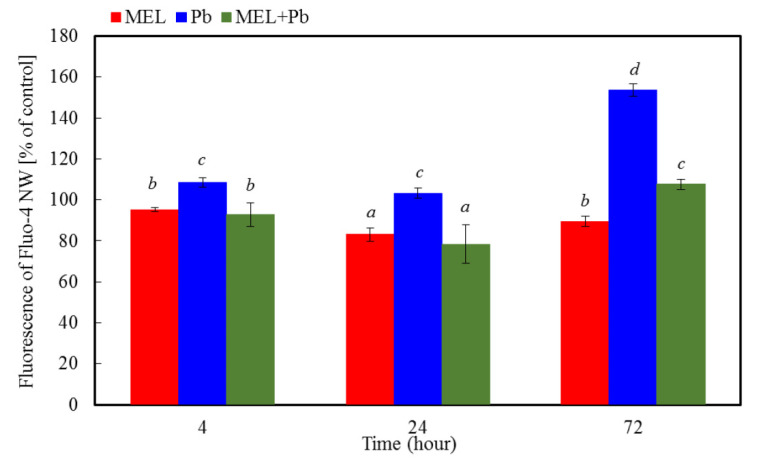
Ca^2+^ concentration in all experimental variants of BY-2 tobacco cells exposed to lead. Cell variants: C—BY-2 cells cultured on LS medium (the control variant); MEL—BY-2 cells cultured on LS medium with 200 nM melatonin added from the beginning of the culture; Pb—BY-2 cells cultured on LS medium with 15 µM Pb^2+^ added on the 4th day of the culture; and MEL+Pb—BY-2 cells cultured on LS medium with melatonin added from the start of the culture and with Pb^2+^ added on the 4th day of culture. The intensity of Fluo-4-NW probe fluorescence was measured: 4, 24 and 72 h after lead treatment. Fluorescence of BY-2 control cells (C) above indicated that moments of the experiment were assumed as 100%. Ca^2+^ concentration ANOVA results: Variant (MEL, Pb, MEL + Pb) F_(2; 18)_ = 157 *p* < 0.00001; Hours of Pb exposure (4, 24, 72) F_(3; 18)_ = 104.7 *p* < 0.00001; and interaction Variant x Hours of Pb exposure F_(4; 18)_ = 30.9 *p* < 0.00001.

The level of the cytoplasm calcium was determined using the fluorescence probe Fluo-4-NW. Figure 2 shows that BY-2 cells primed with melatonin resulted in similar or only slightly decreased levels of the intracellular free calcium in comparison to control cells. Changes in the level of calcium ions were detectable after only 4 h of incubation with lead. A significant rise in the intensity of fluorescence probe was observed, especially during the longer time following lead treatment (72 h), and it was over 50% higher in comparison to control cells. Four and twenty-four hours after heavy metal exposition in the MEL + Pb variant, the intracellular free calcium level was similar to those without lead (MEL variant), whereas after 72 h, the intensity of fluorescence probe was 20% higher than in MEL samples. The strongest evidence that melatonin limited concentration of intracellular calcium ions provided results of comparison between MEL + Pb and Pb variants. Obtained results showed a 20%, 30% and over 40% lower level of the intracellular free calcium in MEL + Pb samples than in Pb ones for 4, 24 and 72 h after lead exposition, respectively.

### 2.3. Changes in Mitochondrial Membrane Potential (ΔΨm)

**Figure 3:**
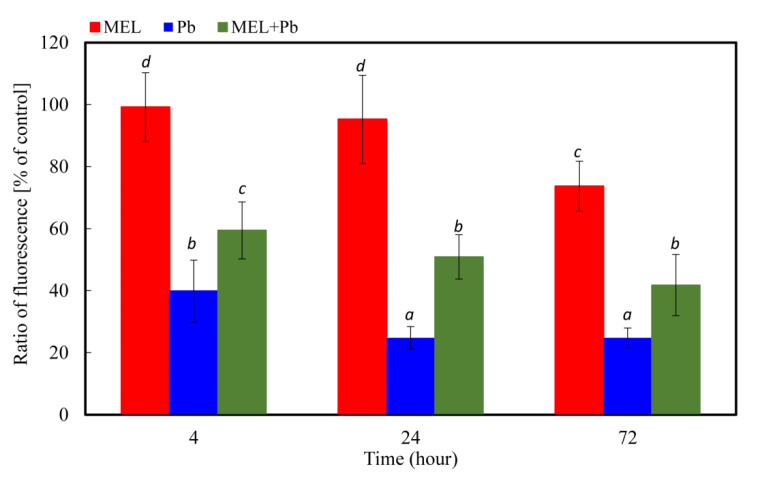
Changes in mitochondrial membrane potential (*ΔΨm*) of BY-2 tobacco cells in conducted experiments. Cell variants: C—BY-2 cells cultured on LS medium – the control variant; MEL—BY-2 cells cultured on LS medium with 200 nM melatonin added from the beginning of the culture; Pb—BY-2 cells cultured on LS medium with 15 µM Pb^2+^ added on the 4th day of the culture; and MEL+Pb—BY-2 cells cultured on LS medium with melatonin added from the start of the culture and with Pb^2+^ added on the 4th day of culture. Fluorescence ratio of JC-1 dimers/JC-1 monomers in BY-2 cells were measured 4, 24 and 72 h after lead treatment. Fluorescence of BY-2 control cells (C) in above indicated moments of the experiment were assumed as 100%. Mitochondrial membrane potential ANOVA results: Variant (MEL, Pb, MEL + Pb) F_(2; 43)_ = 185.3 *p* < 0.00001; Hours of Pb exposure (4, 24, 72) F_(2; 43)_ = 20.6 *p* < 0.00001; and interaction Variant x Hours of Pb exposure F_(4; 43)_ = 2.2 *p* = 0.084.

The changes in mitochondrial membrane potential were detected using the fluorescence dye JC-1. This probe has the unique property of spontaneously forming red-fluorescent dimers under high mitochondrial potential, whereas its monomeric form, prevalent in cells with a low *ΔΨm*, fluoresces in green. Thus, changes in the red/green fluorescence ratio reflect the variation of *ΔΨm*. Figure 3 shows that preincubation of BY-2 cells with melatonin does not reflect a significant reduction in mitochondrial membrane potential. Up to 24 h of incubation, melatonin was observed to decrease the mitochondrial membrane potential only to 73.6 ± 8.07% of control. The lowest level of this parameter was noted for lead-exposed cells (Pb), and our observation indicated that already 4 h after lead treatment, it diminished to 39.85 ± 9.9% of control. Prolonging the incubation time until 72 h resulted in gradual mitochondrial membrane depolarization (24,79 ± 3,19% of control). When the probes were preincubated with melatonin (MEL + Pb variant), the level of *ΔΨm* was about 50% higher in comparison to Pb. Therefore, these results indicate efficiency of melatonin priming against collapse of mitochondrial membrane potential triggered by heavy metal stress (Figure 3).

### 2.4. Cytochrome c Translocation

To further confirm the protective role of melatonin, immunodetection of Cyt c in mitochondrial and cytosolic fractions was examined. The literature data indicate that in plants, similar to in mammals, mitochondria are involved in cell death, and translocation of Cyt c to cytosol seems to play an important role in PCD phenomena. Detection of this protein with an antibody recognizing whole Cyt c molecule was performed in mitochondrial pellet and cytosolic fraction, and subsequently, videodensitometry analysis of both fractions was performed.

**Figure 4:**
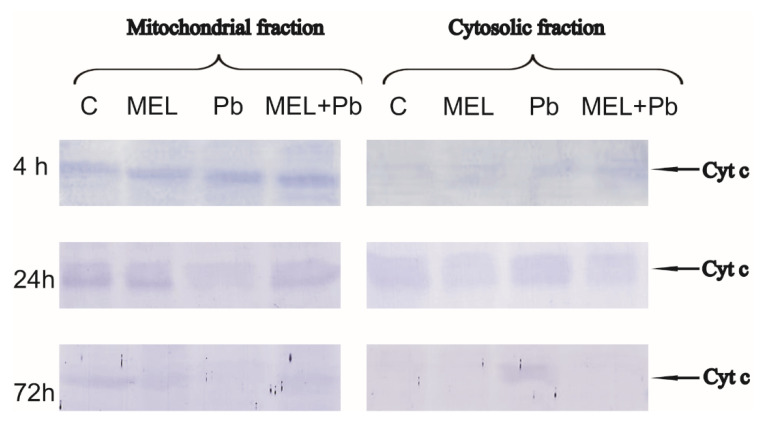
Translocation of cytochrome c protein from mitochondria to the cytosol fraction in all experimental variants: C—BY-2 cells cultured on LS medium, the control variant; MEL—BY-2 cells cultured on LS medium with 200 nM melatonin added from the beginning of the culture; Pb—BY-2 cells cultured on LS medium with 15 µM Pb^2+^ added on the 4th day of the culture; and MEL+Pb—BY-2 cells cultured on LS medium with melatonin added from the start of the culture and with Pb^2+^ added on the 4th day of culture. Cells were exposed to lead for 4, 24 and 72 h. Then, samples were separated by SDS-PAGE and probed with antibodies to cytochrome c by Western blotting.

In all experimental variants in the 4th h after lead treatment, Cyt c was detected mainly in the mitochondrial pellet (Figure 4), despite significant decreasing at this time of the mitochondrial membrane potential. Comparable levels of Cyt c were detected both in mitochondrial pellet and cytosolic fractions of BY-2 cells in control (C) and melatonin-treated samples (MEL and MEL + Pb) in the 24 h after lead administration (Figure 4).

**Figure 5:**
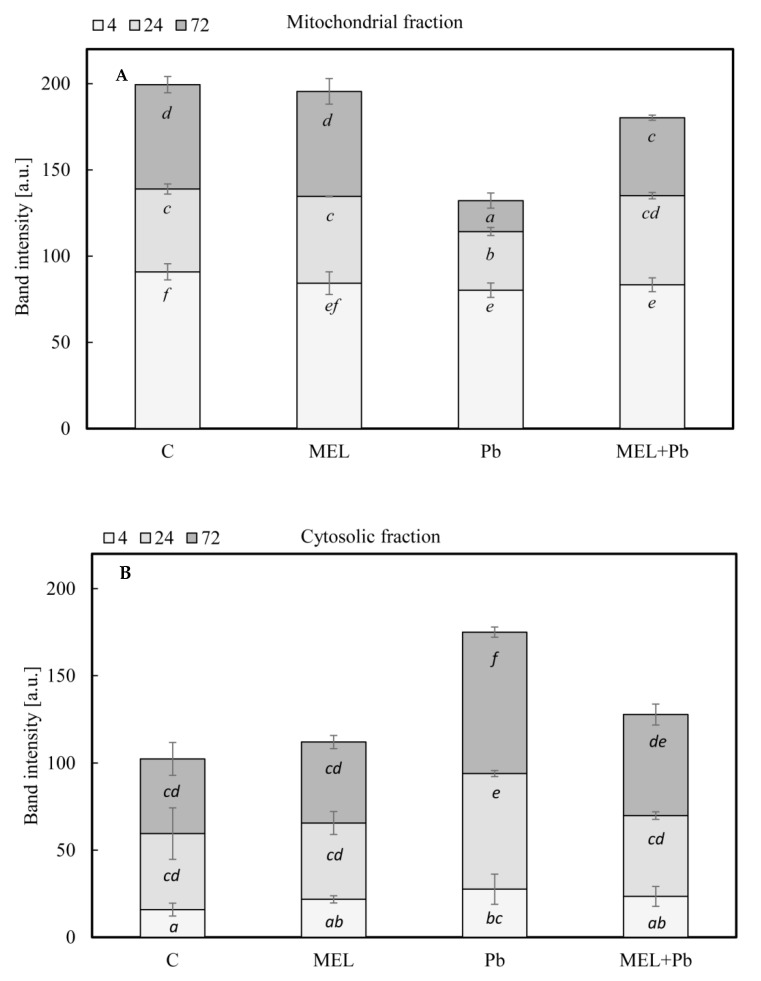
Optical density of cytochrome c in mitochondrial and cytosolic fraction in all experimental variants: **C**—BY-2 cells cultured on LS medium, the control variant; MEL—BY-2 cells cultured on LS medium with 200 nM melatonin added from the beginning of the culture; Pb—BY-2 cells cultured on LS medium with 15 µM Pb^2+^ added on the 4th day of the culture; and MEL+Pb—BY-2 cells cultured on LS medium with melatonin added from the start of the culture and with Pb^2+^ added on the 4th day of culture. Cells were exposed to lead for 4, 24 and 72 h. Then, samples were separated by SDS-PAGE, probed with antibody to cytochrome c by Western blotting and subsequently videodensitometry analysis was perfromed. (**A**) Cytochrome c in mitochondrial fraction ANOVA results: Variant (C, MEL, Pb, MEL + Pb) F_(3; 16)_ = 36.5 *p* < 0.00001; Hours of Pb exposure (4, 24, 72) F_(2; 16)_ = 261.5 *p* < 0.00001; and interaction Variant x Hours of Pb exposure F_(6; 16)_ = 10.1 *p* < 0.0005. (**B**) Cytochrome c in cytosolic fraction ANOVA results: Variant (C, MEL, Pb, MEL + Pb) F_(3; 12)_ = 17.4 *p* < 0.0005; Hours of Pb exposure (4, 24, 72) F_(2; 12)_ = 57.5 *p* < 0.00001; and interaction Variant x Hours of Pb exposure F_(6; 16)_ = 1.3 *p* = 0.323.

In Pb samples, relatively high release of Cyt c from mitochondria to cytosol was observed at this time. The estimated integral density of immunostained polypeptide bands indicated about a 50% higher Cyt c expression in cytosolic fraction in comparison to control (C) or melatonin-primed BY-2 cells (MEL and MEL + Pb) (Figure 5). For BY-2 cells exposed to lead, extension of incubation time to 72 h resulted in almost complete disappearance of Cyt c in mitochondrial pellet and simultaneous accumulation of this protein in cytosolic fraction. Videodensitometric analysis showed increases of the cytosolic Cyt c expression level nearly two times above control and MEL value. Cells treated with melatonin and exposed to heavy metal (MEL + Pb) were characterized by similar Cyt c expression in cytosol, versus lead-untreated ones. However, quantitative analysis showed 25% higher expression of Cyt c in comparison to C and MEL variants and 30% lower Cyt c level relative to Pb (Figure 5). This cytological and molecular evidence demonstrated clearly that melatonin preincubation protected tobacco suspension cells from lead-induced death.

### 2.5. Effect of Pb on Caspase-like Proteolytic Activity

**Figure 6:**
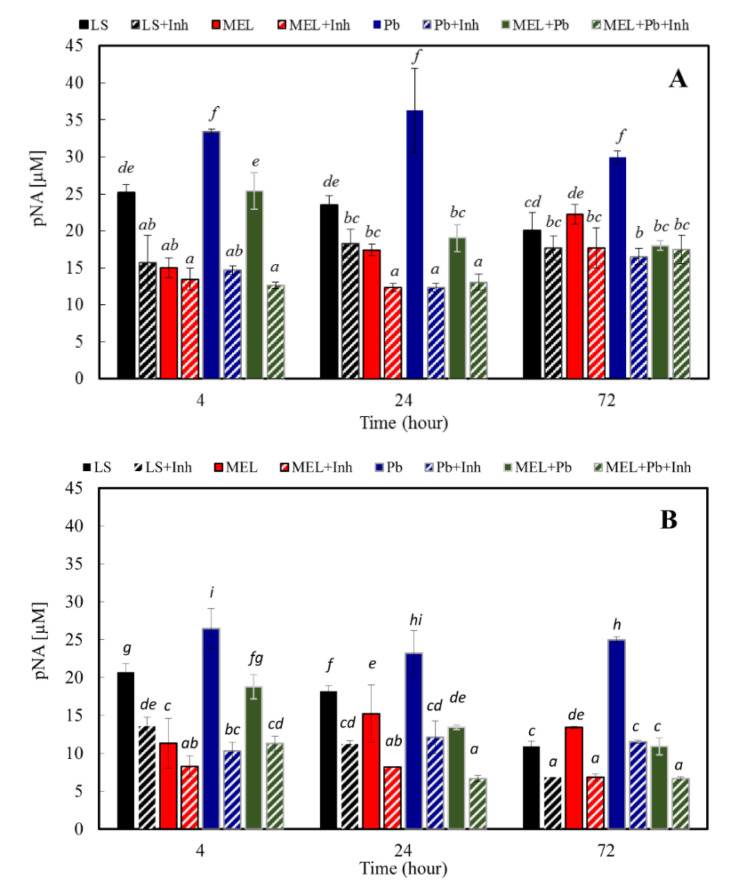
Measurements of caspase-like activities in tobacco cells: (**A**) caspase 9-like activity with (+Inh) or without the caspase-9 inhibitor Ac-LEHD-CHO; (**B**) caspase 3-like activity with (+Inh) or without the caspase-3 inhibitor Ac-DEVD-CHO. Cell variants: C—BY-2 cells cultured on LS medium (the control variant); MEL—BY-2 cells cultured on LS medium with 200 nM melatonin added from the beginning of the culture; Pb—BY-2 cells cultured on LS medium with 15 µM Pb^2+^ added on the 4th day of the culture; and MEL + Pb—BY-2 cells cultured on LS medium with melatonin added from the start of the culture and with Pb^2+^ added on the 4th day of culture. Caspase-like activities were measured 4, 24 and 72 h after lead exposure. (**A**) Caspase-9-like ANOVA results: Variant (LS, LS + Inh, MEL, MEL + Inh, Pb, PB + Inh, MEL + Pb, MEL + PB + Inh) F_(7; 74)_ = 121 *p* < 0.00001; Hours of Pb exposure (4, 24, 72) F_(2; 74)_ = 1.65 *p* = 0.198; and interaction Variant x Hours of Pb exposure F_(14; 74)_ = 9.5 *p* < 0.00001. (**B**) Caspase-3-like ANOVA results: Variant (LS, LS + Inh, MEL, MEL + Inh, Pb, PB + Inh, MEL + Pb, MEL + PB + Inh) F_(7; 87)_ = 180 *p* < 0.00001; Hours of Pb exposure (4, 24, 48) F_(2; 87)_ = 45.5 *p* < 0.00001; and interaction Variant x Hours of Pb exposure F_(14; 87)_ = 13.6 *p* < 0.00001.

The activation of caspase-9 and -3-like proteases has been shown to occur during PCD in several plant systems, suggesting that some forms of plant PCD may have a caspase triggering pathway similar to the animal counterpart [23,49,50,51]. To measure LEHDase (caspase-9-like) and DEVDase (caspase-3-like) activities, total protein extracts from BY-2 suspension cells in all experimental variants were incubated with the synthetic tetrapeptide LEHD-pNA and DEVD-pNA, a specific substrate of caspase-9 and -3, respectively. Addition of the substrate resulted in a signal caused by the cleavage of the chromophore pNA from the labelled substrate. LEHDase and DEVDase activity was monitored after 4, 24 and 72 h of cell treatment with lead. Cells primed with melatonin and not exposed to heavy metal demonstrated lower or similar to control caspase-9 and -3-like proteolytic activity in all analysed times of culture. However, extremely high caspase-9-like activity was noticed in Pb variants, and it was about 35%, 55%, and 50% higher in comparison to C. We found the inhibition of LEHDase activity in cells incubated with melatonin and treated with lead (MEL + Pb). The strongest evidence was especially visible 24 and 72 h after heavy metal incubation, and for MEL + Pb samples, caspase-9-like proteolytic activity was over 90% lower than in Pb ones (Figure 6A). This investigation confirms that melatonin, to a large extent, is able to block/reverse the cytotoxic lead influence and protect tobacco cells against death caused by heavy metal. Similar effects were obtained for DEVDase. Figure 6B shows that BY-2 cells incubated with lead had enhanced caspase-3-like activities in all analysed time points after lead application. Nevertheless, the biggest differences in caspase-3-like activity between control and lead-treated cells (Pb) were observed at the end of culture (72 h after heavy metal application) and it was 10.9 versus 24.9 µM pNA for C and Pb, respectively. Usage of melatonin resulted in a reduction of caspase-3-like activity to a level similar to lead-untreated cells. Both caspase-like protease activities were significantly decreased by caspase inhibitors specific for each caspase: Ac-LEHD-CHO (for caspase-9) and Ac-DEVD-CHO (for caspase-3) (Figure 6A,B), validating presented data. These results suggest increasing caspase-like activity occurred during PCD of the tobacco suspension cells.

### 2.6. Determination of Melatonin Levels

To find out whether tobacco cells are able to actively absorb melatonin from culture media, the contents of this indoleamine in cell lysates were determined by HPLC-MS in *lag, log* and stationary phase of growth.

Generally, tobacco cells have melatonin content on a low level (Table 1). In not exposed to melatonin cells (C and Pb), the level of this indoleamine slightly increased from zero (the 1st day of culture) to ~1 ng/g_FW_ (on the last day). In comparison with the melatonin-primed cells (MEL and MEL + Pb) BY-2 cells are able to synthesize endogenous melatonin, but in extremely low levels. Moreover, in lead-treated cells (Pb), the level of endogenous melatonin in comparison to the control (C) cells was about 30% lower at the end of the culture period (7th day). The concentrations of melatonin drastically increased in the cells exposed to melatonin during the whole period of culture (Table 1). Obtained data indicated that BY-2 cells absorbed it gradually from the medium. Cells exposed to heavy metal stress condition and primed with melatonin (MEL + Pb) absorbed this indoleamine about 20% more intensively than MEL cells.

## 3. Discussion

Plant cells have the ability to self-destruct in a controlled manner, called programmed cell death. Unlike death as a consequence of physical damage, which is not controlled by the cell itself, PCD is genetically regulated. PCD is a versatile tool for plants to cope with various situations or needs. Within plant systems, PCD falls within two broad categories, developmentally regulated and environmentally induced. In development, tracheary elements for example, undergo PCD to form the xylem; moreover, deletion of the embryonic suspensor, anther dehiscence, leaf and flower senescence or leaf morphogenesis also belong to developmentally regulated PCD [52,53,54]. PCD also plays an important role in plants exposed to a broad range of biotic and abiotic stress stimuli such as pathogen–plant reactions, UV radiation, heat shock and heavy metals, e.g., aluminium, coper, cadmium or lead [9,54,55,56]. Our previous studies indicated that lead-induced PCD manifested on the cytological level by ROS accumulation leading to DNA fragmentation and cell death [10]. 

This report regards the involvement of mitochondria in lead-induced cell death and the protective effect of melatonin on in vitro plant suspension cells exposed to lead stress.

At the first part of this study, cell proliferation and viability under optimal (C and MEL) and heavy metal stress condition (Pb and MEL + Pb) were investigated. *Nicotiana tabacum* BY-2 cells cultivated on LS medium supplemented with melatonin (MEL) did not significantly change cell development profiles. Both levels of cell proliferation as well as their mortality were similar to non-treated control cells cultivated under optimal conditions. Positive effects of melatonin were visible during lead stress. Proliferation of cells pre-treated with melatonin under heavy metal stress condition (MEL + Pb) was only slightly worse in comparison to lead-unstressed variants (C and MEL), whereas in Pb probes, a significantly lower proliferation level was observed (about 50% of control cells). Conversely, the protective role of melatonin was evaluated by cell mortality in all experimental samples. We expect that the number of dead cells in Pb variant increased drastically during heavy metal stress. However, we did not suppose that such high viability would be obtained in a melatonin pre-incubated cell variant exposed to heavy metal (MEL + Pb), where its viability was about 80% higher than in Pb cells. Our results are in line with many previous results, which also indicate that positive effects of plant melatonin treatments do not appear under optimal conditions, but this indoleamine can act as a factor fortifying cells against potentially stress conditions before it appears [9,34].

It is well established that melatonin exerts the antiapoptotic action in various animal cells, and the basis of this action is associated with its antioxidant properties [57,58,59,60]. Although the significance of melatonin for plant life is still being intensively studied, the current knowledge is mainly focused on the role of melatonin as an antioxidant factor, which takes part in direct scavenging of ROS and RNS [61], acceleration of antioxidant enzymes activity [33], protection against oxidative damage leading to cell death [62], and synergistic actions with other antioxidants [63]. Melatonin is also a growth promoter that limits cell death by inhibition of machinery, leading to DNA laddering [10,64] or by influence on BI-1 protein, which overexpression in plants improved cell survival against elicitors of plant fungi [65,66] or heavy metals [9]. To elucidate the mechanism of melatonin action against PCD limitation induced by lead, we tested the main biochemical hallmarks of cell death, such as the changes in intracellular calcium, changes in mitochondrial membrane potential, the level of Cyt c and LEHDase and DEVDase activity. Mitochondrial Ca^2+^ overload is, in animals, one of the pro-apoptotic ways to induce the swelling of mitochondria, with perturbation or rupture of the outer membrane, and in turn, the release of some mitochondrial apoptotic factors, such as Cyt c into the cytosol [67]. In plant cells, it is also widely recognized that intracellular Ca^2+^ is an important regulator of PCD [26,27,68]. Our research indicated that BY-2 cells primed with melatonin demonstrated similar or only slightly decreased levels of intracellular free calcium in comparison to control cells. However, changes in the intensity of fluorescence were detectable after only 4 h of incubation with lead. A significant rise in level of calcium ions was observed, especially during the longer time following lead treatment (72 h), and it was over 50% higher in comparison to the control cells. Simultaneously, in the MEL + Pb variant, the intracellular free calcium level was similar to those without lead. The strongest evidence that melatonin limited the concentration of intracellular calcium ions provided results of comparison MEL + Pb vs. Pb samples. Obtained results showed time-dependent reduction of the intracellular free calcium in MEL + Pb cells vs. Pb ones. Conversely, Kacprzyk et al. [69] showed that the activation of the PCD pathway depends on one initial Ca^2+^-dependent trigger, rather than a series of calcium influxes throughout the death process. It was also demonstrated that blocking the extracellular Ca^2+^ influx was accompanied by a corresponding decrease in the level of early DNA fragmentation, which is another marker of PCD. Results obtained in the laboratory of Li et al. [26] explained the time discrepancies in Ca^2+^ level and DNA fragmentation. According to them, intracellular calcium ions could be divided into cytoplasmic calcium ([Ca^2+^] cyt) and nuclear calcium ([Ca^2+^] nuc. The rise of [Ca^2+^] cyt could promote the production of ROS in cells, and the PCD process is induced by the gradually increasing concentration of ROS. However [Ca^2+^] nuc is an important factor triggering nuclease and serine protease activity in the nucleus [26]. In addition, the experimental results implied that the increase of [Ca^2+^] nuc concentration is necessary for the occurrence of PCD, but [Ca^2+^] nuc would only be induced until the intracellular ROS has gradually accumulated to a higher level. These data seem to support the hypothesis of Collazo et al. [70] that the NO/ROS ratio may induce a set of defence responses, including cleavage of an inhibitor of caspase-activated DNAse (ICAD) [70,71]. It is also in line with our previous report that DNA fragmentation was observed before Cyt c translocation to cytosol [10]. The mechanisms of Cyt c release inhibition by melatonin are still unknown. In animals, Suofu et al. [72] delineated a mitochondrial mechanism contributing to the protective action of melatonin against cell death. The high-affinity of melatonin and 2-[125I]-iodomelatonin for binding to melatonin receptors located in the outer membrane of mitochondria was established. Further described were mitochondrial melatonin type 1 receptors responding to melatonin by activating heterotrimeric G proteins located in the intermembrane space, blocking adenylate cyclase activity and inhibition of Ca^2+^ dependent, stress-mediated Cyt c release, and in consequence, damping of caspase activation. Our results revealed gradual mitochondrial membrane depolarization after lead treatment. However, when the probes were preincubated with melatonin (MEL + Pb), the level of *ΔΨm* was about 50% higher in comparison to Pb alone. It was compatible with data of Cyt c translocation from mitochondria to cytosol. Western blot and subsequent videodensitometric analysis showed increases of the cytosolic fraction of Cyt c expression in lead-treated BY-2 cells, nearly two times above control and MEL values. Cells treated with melatonin and exposed to heavy metal (MEL + Pb) were characterized by similar Cyt c expression in cytosol to lead-untreated ones. In mammals, following the outflow of Cyt c from mitochondria apoptosome, a heptameric structure consisting of Apaf-1 protein, ATP, Cyt c, and procaspase-9 is formed, and this key factor leads to the activation of caspase cascade and cell death. While caspase activities have been detected in plants, sequences similar to animal caspases are not present in plant genomes. The metacaspases with weak structural similarity to caspases are likely involved in PCD [73,74], but do not execute caspase-specific proteolytic activity, recognizing substrates with either lysine or arginine instead of aspartate [75]. Other plant proteases with limited similarity to animal caspases display caspase-like activities and are involved in diverse types of PCD. In particular, vacuolar processing enzyme (VPE), also called legumain, is responsible for caspase-1 activity in plants [76]. In turn, the 20S proteasome, composed of many α and β subunits, executes caspase-3 activity in response to biotic stress [77]. Wang et al. [23] observed activation of LEHDase and DEVDase during programmed cell death induced by sulphated lentinan in BY-2 cells, whereas Tran et al. [74] showed an increase in cascade of caspase-1, -3, -4, -6 and -8-like (excluding caspase-9-like) activities in the endosperm fraction during early development of barley caryopsis; however, in the maturating endosperm, an increase of all above caspase-like activities, including of caspase-9-like, was detected.

Our results demonstrated LEHDase and DEVDase activity in all analysed time points after lead exposition. However, cells primed with melatonin and lead-untreated (MEL) demonstrated lower or similar to control caspase-9 and -3-like proteolytic activity. Obtained data revealed about a 50% increase in caspase-9-like activity after lead administration in comparison to control. Conversely, incubation of BY-2 cells with melatonin before heavy metal exposition (MEL + Pb) resulted in the inhibition of LEHDase and DEVDase activity. This investigation confirms that melatonin, to a large extent, is able to block/reverse the cytotoxic lead influence and protect tobacco cells against death caused by heavy metal, and it seems that the mechanism of this inhibition is achieved via mitochondria.

Several data collected in the review by Reiter et al. [47] show that melatonin is an important mitochondria-targeted antioxidant. This indoleamine not only prevents oxidative stress, but it also enhances mitochondrial antioxidant enzymes, improving the efficiency of electron transport in the mitochondrial chain as well as preventing the PCD pathway [46,48,78]. In transgenic *Arabidopsis* lines ectopically expressing *MzSNAT5* gene, significantly higher melatonin production was observed, and surprisingly, synthesis of this indoleamine took place in mitochondria [48]. Moreover, in mammals, new transport systems, namely GLUT/SLC2A and PEPT1/2, were shown to have an active role in facilitating melatonin transport across membranes. Furthermore, the localization of these two transport systems in the mitochondria strongly suggests the participation of both in-mitochondrial transport of melatonin [46,79,80].

Results of our research indicate that tobacco BY-2 cells were able to synthesize various, small amounts of this indoleamine, depending on the phase of growth as well as to absorb it actively from the medium. Availability of exogenous melatonin allowed BY-2 cells to uptake its large quantities throughout the culture period. Its levels at the end of culture were over 30 to 60 times higher in MEL and MEL + Pb in comparison to C and Pb, respectively. The similar effects were observed by Kołodziejczyk et al. [81] in the case of cucumber and corn seeds, which were osmo- or hydroprimed with exogenous melatonin—corn seeds absorbed great quantities of this indoleamine proportional to its concentration applied during priming. Moreover, our previous data also revealed that, in sugar-starved BY-2 cells exposed to melatonin, the level of this indoleamine was much more higher than in sugar-starved but melatonin-untreated cells [41]. It could suggest that BY-2 cells absorb exogenous melatonin more readily and in larger quantities to counteract stress-induced cell death.

In conclusion, we indicate that melatonin successfully reverses the toxic influence of lead and protects plant cells against death. However, taking the newest literature data and our results into account, it seems probable that many PCD-protected functions could be explained on the basis of the melatonin presence in mitochondria; for this purpose, further studies of proteins and gene expression (involved in plant’s PCD) in the investigated experimental model are planned.

## 4. Materials and Methods

### 4.1. Cell Culture and Growth Conditions

BY-2 suspension cells were routinely propagated and cultured at 25 °C. From the stationary growth phase (day 7th) of the base culture, 2 mL of cell suspension was passaged into the fresh LS medium as a control (C) and LS with 200 nM melatonin (MEL). The optimal dose of melatonin was chosen experimentally. In the middle of the logarithmic phase of growth (day 4th) Pb(NO_3_)_2_ was added to LS (Pb) and LS with melatonin (MEL + Pb) media to the final Pb^2+^ concentration of 15 µM. The applied concentration of lead was chosen after measurement of LC_50_ on the 7th day. Thus, the experiments were performed in four variants: (i) C: BY-2 cells cultured under optimal conditions on LS medium; (ii) MEL: BY-2 cells cultured on LS medium supplemented with melatonin from the start of new culture; (iii) Pb: BY-2 cells cultured on LS medium with Pb^2+^ added on the 4th day of culture; and (iv) MEL+Pb: BY-2 cells cultured on LS medium with melatonin added from the start of culture and stressed with Pb^2+^ added on the 4th day of culture. The cultures were maintained to the 7th day (stationary phase of the control cell growth).

### 4.2. Determination of Cell Growth and Viability

The cell number was determined with the use of a Fuchs–Rosenthal haemocytometer under a light microscope Olimpus CX-31 equipped with MicroScan v.15. digital system of image analysis; additionally, the number of dead cells was assessed after selective staining with methylene blue. Living cells do not take up the stain and retain their natural colour, whereas damaged cells are stained blue, as they are unable to keep the methylene blue from penetrating their membranes. The number of cells and their viability were analysed every experimental day.

### 4.3. Intracellular Calcium Ions Detection

Intracellular calcium level was determined using the fluorescent probe Fluo-4NW, which is able to cross the plasma membrane of living cells. After entering the cell, Fluo-4NW is converted by cytosolic hydrolases to the active form, having the possibility of binding of calcium ions. As a result of joining of calcium ions, the probe emits fluorescence (λ_em_ = 538 nm) after excitation with light of wavelength 485 nm. Tobacco BY 2 cells in all experimental variants were plated in 96-well black fluorometric plates (2 mg cells/well) and washed with PBS in order to eliminate sources of baseline fluorescence. Finally, a dye loading solution (Fluo-4-NW dye, 4-[(Dipropylamino)sulfonyl] benzoic acid (Probenecid) was used to inhibit extrusion of the indicator out of the cell by organic anion transporters. Hanks’ balanced salt solution (HBSS), 20 mM N-(2- hydroxyethyl)piperazine-N’-(2-ethanesulfonic acid) buffer solution (HEPES) was added in a volume of 100 μL per well and incubated for 30 min in the total darkness at 37 °C, and then for the next 30 min at room temperature. The measurement was performed on Fluoroskan Ascent FL microplate reader (Labsystems, Sweden) using 494 nm excitation and 516 nm emission wavelengths.

### 4.4. Mitochondrial Membrane Potential (ΔΨm)

Cells with all experimental variants were seeded into black 96-well titration microplates and incubated with 5 µM JC-1 in HBSS for 30 min at 37 C in the dark. JC-1 is a fluorescent carbocyanine dye which accumulates in the mitochondrial membrane in two forms (monomers or dimers), depending on mitochondrial membrane potential. JC-1 monomers show maximum fluorescence excitation and emission at 485 and 538 nm wavelengths, respectively. Negative potential of the inner mitochondrial membrane facilitates the formation of dye aggregates, which results in the shift of JC-1 monomer fluorescence towards red light (λex = 530 nm to λem = 590 nm) [82]. Thus, the measurement of the JC-1 dimer to monomer fluorescence ratio is a convenient and reliable method for the estimation of changes in mitochondrial membrane potential. The fluorescence of both JC-1 monomers and dimers was measured with a Fluoroskan Ascent FL microplate reader (Labsystems, Stockcholm, Sweden). Prior to fluorescence measurements, the cells were washed twice with HBSS to remove the dye that may have adsorbed to the plastic microplate wells and disturbed the measurements. The results are expressed as a ratio of dimer to monomer fluorescence in relation to the control fluorescence ratio, taken as 100%.

### 4.5. Cell Fractionation

Fractionation of cells was performed using the digitonin method according to Ganju and Eastman, with modification of Kobylińska et al. [83,84]. In all experimental variants, the cells were washed twice with PBS and next permeabilized for 30 min in a buffer containing: 1 mM NaH_2_PO_4_, 8 mM Na_2_HPO_4_, 75 mM NaCl, 250 mM sucrose, digitonin (0.05% of cells weight), 20 µL/g cells 1 mM phenylmethylsulfonyl fluoride (proteases inhibitor), and cocktail of enzymes for cell wall lysis (CelLytic Sigma). Cell homogenate was obtained by centrifugation at 3000× *g* for 1 min. at 4 °C to remove cell debris. The cleaned homogenate after centrifugation at 12,000× *g* was divided into two fractions: the supernatant was removed as the cytosolic fraction and the pellet (mitochondrial fraction) was resuspended in the above buffer (without digitonin). To both fractions, sufficient volumes of Laemmli sample buffer supplemented with 10% β-mercaptoethanol were added [85], and the mixtures were boiled for 5 min.

### 4.6. Western Blot Analysis

Fractionated BY-2 cell lysates (50 μg of proteins) were electrophoretically separated by sodium dodecyl sulphate polyacrylamide gel electrophoresis (SDS-PAGE) on 15% gel [85] and transferred to Immobilon P^SQ^ at the voltage of 20 V overnight at 4 °C, according to Towbin et al. [86]. After blocking in 3% nonfat dry milk in TBST (10 mM Tris-HCl, pH 7.5, 150 mM NaCl, 0.05% Tween-20) for 60 min, the membranes were incubated with primary antibodies specific to Cyt c in TBST in a cold room overnight. Subsequently, the membranes were washed several times in TBST and incubated with appropriate secondary antibodies conjugated with alkaline phosphatase (Sigma Chemical Co.) in TBS for 2 h at room temperature. Next, the membranes were washed several times with TBST. The proteins were visualized by incubation with the substrate solution (0.33 mg/mL of nitro blue tetrazolium, 0.17 mg/mL of 5-bromo-4-chloro-3-indolyl phosphate in 100 mM Tris-HCl, pH 9.5, 100 mM NaCl and 5 mM MgCl_2_), prepared according to Leary et al. [87] and subsequently, videodensitometry analysis was performed (GelAnalyzer 2010a.).

### 4.7. Measurements of Caspase-like Activities

Caspase-9 (LEHDase) and caspase-3-like (DEVDase) activities were determined using the Caspase-9 and Caspase-3 Colorimetric Activity Assay Kits (EMD Millipore, Germany), respectively, according to the manufacturer’s instructions. The assays are based on spectrophotometric detection of the chromophore pNA after the caspase-dependent cleavage from the labelled substrates LEHD-*p*NA or DEVD-*p*NA by active caspase-9 or -3, respectively. Cytosolic protein extracts from tobacco BY-2 cells after cell wall lysis were incubated for 2 h at 37 °C with LEHD-*p*NA or DEVD-*p*NA in the presence or absence of 3 μM caspase inhibitors Ac-LEHD-CHO (for caspase-9) or Ac-DEVD-CHO (for caspase-3) before spectrophotometric quantification of the free pNA (λ = 405 nm). There were three independent replicates.

### 4.8. Melatonin Determination

Melatonin was extracted according to the modified methods of Guerrero et al. and Hernandez- Ruiz et al. [88,89]. Its concentration was measured on the 1st, 4th and 7th day, which corresponds to *lag, log* and the stationary phases of growth. After filtration and separation of the cells from the medium, concentrations of melatonin in the extracts were determined using high-performance liquid chromatography (HPLC-MS/MS). For extraction, 5 g of fresh weight of the cells was homogenized with 5 mL of 50 mm sodium phosphate buffer (pH 8.0) containing 1 mm EDTA and 5 μM butylated hydroxytoluene (BHT) as an antioxidant. The homogenate was maintained for 15 h at room temperature in darkness with minimal shaking in order to ensure complete extraction of melatonin.

Then, it was centrifuged at 1500× *g* for 10 min at 5 °C. Initial purification consisted of two steps by solvent partitioning using ethyl acetate and 50 mm sodium phosphate buffer (first at pH 8.0 and second at pH 3.0). The two organic phases were evaporated together under vacuum. Dry residue was re-dissolved in 1 mL of mobile phase, filtered through Supelco ISO-Disc filters (PTEF-4—2.4 mm × 0.2 m; Supelko, Bellefonte, PA, USA), and frozen at −70 °C until HPLC-MS analysis. The purified extract was subjected to HPLC-MS/MS analysis using an Agilent 1200 LC System coupled with AB Sciex 3200 QTRAP mass detector equipped with TurboSpray Ion Source (ESI). Each sample was injected onto Agilent SB-C18 column.

### 4.9. Statistical Analysis

The data represent the means ± standard deviation (±SD). The data were analysed using STATISTICA v.10.0_MR1_PL (StatSoft) software. One-way or two-way analysis of variance (ANOVA) and then post hoc Duncan multiple range test were carried out to find the significant differences at *p* < 0.005 in each experiment. Statistically significant differences were marked as different small letters on graphs and in the table.

## Figures and Tables

**Table 1:** Melatonin concentration (ng_MEL_/g_FW_) in homogenates of BY-2 cells in in all experimental variants: C—BY-2 cells cultured on LS medium—the control variant; MEL—BY-2 cells cultured on LS medium with 200 nM melatonin added from the beginning of the culture; Pb—BY-2 cells cultured on LS medium with 15 µM Pb^2+^ added on the 4th day of the culture; and MEL+Pb—BY-2 cells cultured on LS medium with melatonin added from the start of the culture and with Pb^2+^ added on the 4th day of culture. Measurements were conducted 4, 24 and 72 h after Pb treatment.

Time (h)	BY-2 Cell Variants			
	C	MEL	Pb	MEL+Pb
4	0.00 ± 0.00 *a*	6.68 *b* ± 0.13 *b*	0.00 ± 0.00 *a*	6.68 ± 0.13 *b*
24	0.73 ± 0.02 *a*	15.08 *c* ± 1.59 *c*	0.73 ± 0.02 *a*	15.08 ± 1.59 *c*
72	0.95 ± 0.04 *a*	33.24 *d* ± 3.19 *d*	0.68 *a* ± 0.04 *a*	40.75 *e* ± 3.25 *d*

Melatonin ANOVA results: Variant (C, MEL, Pb, MEL + Pb) F_(3; 59)_ = 831.9 *p* < 0.00001; Hours of Pb exposure (4, 24, 72) F_(2; 59)_ = 600.1 *p* < 0.00001; and interaction Variant x Hours of Pb exposure F_(6; 59)_ = 190.8 *p* < 0.00001.

## Data Availability

Not applicable.
